# Reappraisal Value of a Modified Rotational Atherectomy Technique in Contemporary Coronary Angioplasty Era

**DOI:** 10.1155/2020/9190702

**Published:** 2020-01-23

**Authors:** Haojian Dong, Daisuke Hachinohe, Zhiqiang Nie, Yoshifumi Kashima, Jianfang Luo, Takuya Haraguchi, Hidemasa Shitan, Tomohiko Watanabe, Yutaka Tadano, Umihiko Kaneko, Takuro Sugie, Ken Kobayashi, Daitaro Kanno, Morio Enomoto, Katsuhiko Sato, Tsutomu Fujita

**Affiliations:** ^1^Department of Cardiology, Vascular Center, Guangdong Cardiovascular Institute, Guangdong Provincial Key Laboratory of Coronary Heart Disease Prevention, Guangdong Provincial People's Hospital, Guangdong Academy of Medical Sciences, Guangzhou, China; ^2^Sapporo Heart Center, Sapporo Cardio Vascular Clinic, Sapporo, Japan; ^3^Department of Epidemiology, Guangdong Cardiovascular Institute, Guangdong Provincial People's Hospital, Guangdong Academy of Medical Sciences, Guangzhou, China

## Abstract

**Objectives:**

To introduce a modified rotational atherectomy (RA) procedure and investigate the early and midterm outcomes of the RA-facilitating diversified percutaneous coronary intervention (PCI) in a large group of aged patients with higher cardiovascular risk.

**Background:**

Previous studies about the outcomes of RA were limited with small sample size and low-risk population.

**Methods:**

Between January 2013 and November 2015, 1169 consecutive patients treated with modified RA-facilitated PCI were retrospectively enrolled, including de novo calcified lesions and in-stent restenosis. Patients were regularly followed up for at least 1 year. Major adverse cardiac events (MACE) were analyzed for all participants by different strategies. Cox regression analysis was performed to identify risk factors for the events.

**Results:**

The median age of patients was 75 years, with 11.7% of patients on maintenance hemodialysis. Most lesions (99.9%) were complex (American Heart Association type B2/C), and 68.3% were treated with RA + drug-eluting-stent (DES). Successful angiography was achieved in 97.8% cases, with 1.7% (20/1169) experiencing coronary perforation (including guidewire perforation). The incidence of MACE was 20.5% and 26.8% at 1-year and 2-year follow-up and were mainly driven by target lesion revascularization (TLR) (10.3% and 12.5%, respectively). The strategy of RA + DES had the lowest 2-year MACE, compared with the RA + drug-coated balloon and RA + plain old balloon angioplasty (14.5%, 30.5%, and 26.0%, respectively).

**Conclusions:**

The modified RA technique is a safe and effective tool in the contemporary PCI era, even in high-risk patients. The TLR rate was relatively high but acceptable in such complex lesions.

## 1. Introduction

In contemporary PCI practice, rotational atherectomy (RA) is considered as an adjunctive tool for the management of fibrotic or heavily calcified coronary lesions by “differential cutting” and “orthogonal displacement of friction” [1–3]. RA has been utilized in all main percutaneous coronary intervention (PCI) eras with the employment of plain old balloon angioplasty (POBA), bare-metal stents (BMS), drug-eluting stent (DES), and drug-coated balloon (DCB) [4, 5]. RA presented favorable effects during complex PCI in cumulative studies, such as the STRATAS [6], ROTATE [7], ROTAXUS [8], and DCB [9] studies.

However, the volume of RA was reported to vary widely in these two decades and between individual centers, ranging from 0.6%–20% [10, 11]. In addition, the use of RA showed a trend of progressive decline due to the advent of the DES, the disappointingly high restenosis rate, and lack of impact on major adverse cardiac events (MACE) [12, 13]. In fact, these studies mainly investigated the effect of RA in patients with low or moderate risk [8, 14], including relatively young age (median 70 years) and less complex lesions (50–90%). Nevertheless, in Japan, the trend of social aging has led to patients with more complex PCI situations, severe calcifications, and a high proportion of diabetes and comorbid hemodialysis, while studies evaluating the effect of RA in this group of patients with high risk are rare.

On the other hand, utilization of RA never stopped in daily practice. In particular, with advancement in PCI devices and perioperative preparation with new drugs, the contemporary RA technique has also evolved in the past two decades yielding improved PCI performance [1, 14]. Studies have indicated that the operators' skills and performance in RA are vital for the prognosis [6, 7]. Therefore, further knowledge and education for performing RA in contemporary clinical practice are needed.

In the past 5 years, the Sapporo Cardio Vascular Clinic (SCVC) has modified the RA technique and achieved favorable results for complex PCI [15]. With the purpose of better assessing the performance of RA, this study introduced the use of the modified RA technique, and retrospectively evaluated the “real-world” outcomes of the technique in a large group of patients with high risk in the contemporary coronary angioplasty era.

## 2. Materials and Methods

### 2.1. Study Population

Between January 2013 and November 2015, data of a consecutive series of patients treated with RA-facilitated PCI were retrospectively collected from SCVC. The indications for RA included (1) moderate-to-severe superficial calcification lesions observed on intravascular ultrasound (IVUS)/optical coherence tomography (OCT) images or linear calcium density images on both sides of the target lesion visible under fluoroscopy; (2) calcified lesions making the passage of imaging probes difficult, where inadequate stent delivery or expansion can be expected; (3) calcified ostial and true bifurcating lesions; (4) chronic total occlusion (CTO) lesions, in which the guidewire has been correctly positioned but low-profile balloons cannot be advanced; and (5) selected cases of diffuse in-stent restenosis. The decision to perform RA and PCI was at an experienced and high-volume operator's discretion. Cases with stand-alone RA, bailout use of suboptimally expanded stents, rotablation at the same PCI stage, and incomplete follow-up data were excluded. Clinical follow-up was conducted regularly at least once within 2-years in outpatient clinics by operators. Coronary computed tomography angiogram was routinely performed within 1 year, and a coronary angiogram was suggested when clinically driven (such as new-onset patient symptoms, evidence of cardiac ischemia, or high index of clinical suspicion for significant coronary disease). Informed consent was obtained from each patient. Data were collected using a standardized case report form to record demographic and clinical characteristics and procedural and follow-up data.

### 2.2. Procedure

Immediately before the intervention, patients received intra-arterial or intravenous heparin (70–100 IU/kg) to maintain the activated clotting time over 250 sec. Dual antiplatelet therapy with aspirin and thienopyridine (ticlopidine, 100 mg twice daily or clopidogrel, 75 mg once daily) was used at least one year after PCI and continued as long as possible with aspirin or another type of antiplatelet drug. RA was performed using a Rotablator™ (Boston Scientific, Natick, MA, USA). In accordance with the Japanese insurance policy, a maximum of two burrs were used when required. The choice of stenting (DES/BMS), DCB, or POBA alone was dependent on the operator's judgment.

**Figure 1:**
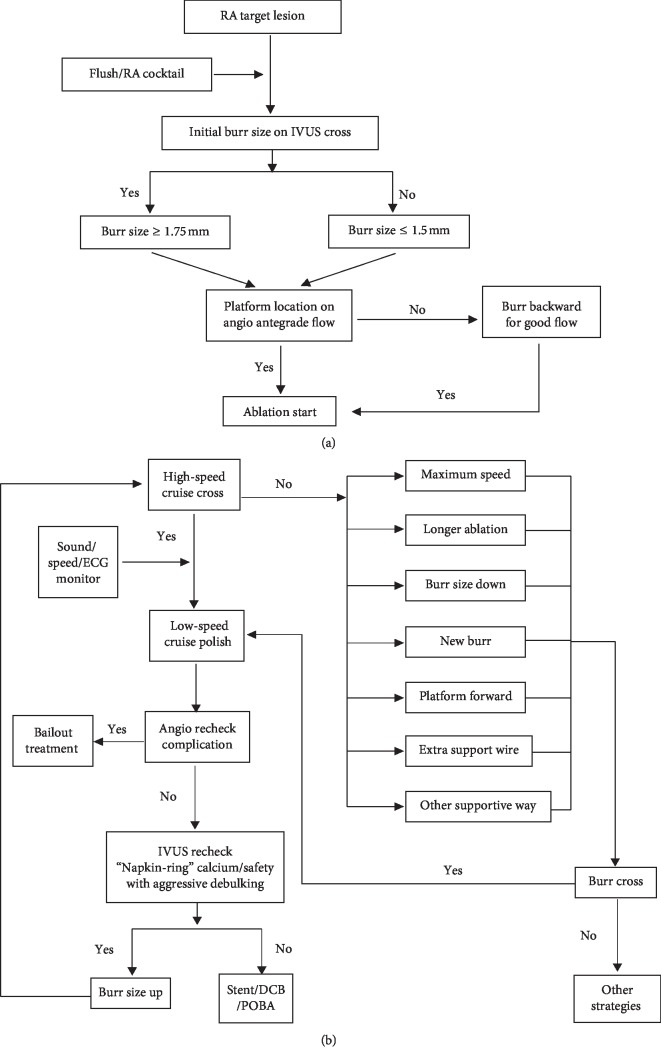
(a) Rotational atherectomy preparation. (b) Rotational atherectomy (RA) algorithm. DCB, drug-coated balloon; ECG, electrocardiogram; IVUS, intravascular ultrasound; POBA, plain old balloon angioplasty; RA, rotational atherectomy.

Concordant with the general principle of RA performance [1, 14], the modified RA technique employed in SCVC is shown in Figures 1(a) and 1(b). It was briefly described as follows: (1) the platform was located immediately proximal to the lesion. In diffuse and distal lesions, the “platform” would move forward with segment after segment ablation for more support. Each platform was confirmed by contrast injection to guarantee good antegrade flow with the burr present. (2) Together with RA-cocktail (saline 1000 ml, heparin 3000 U, verapamil 1.25 mg, and nitroglycerin 5 mg) shower, concomitant intracoronary flush-cocktail (Ringer's lactate 500 ml, nicorandil 12 mg, and nitroglycerin 2 mg) injection from the guiding catheter by mechanical injection was used to cool the Rotablator turbine and clear the pulverized tissue through the coronary microvasculature. (3) The initial burr size depended on whether the IVUS probe could cross the lesion as well as on the vessel diameter. If the IVUS probe could cross the lesion, ≧1.75 mm burr would be initially used. Otherwise, ≦1.5 mm would be used. A “Step-up” strategy would be used for sufficient ablation if the “napkin ring” of calcium remained, and a larger burr would be used for even more aggressive ablation when safe IVUS images were observed. (4) The burr was advanced with slow cruising motion and short run times (15–20 s) and running at “high” speed (160,000–220,000 rpm) to cross and “low” speed (140,000–160,000 rpm) to polish, with an exclusive technician monitoring the rpm versus time during ablation. (5) IVUS/OCT was mandatory for lesions before and after evaluation. The distinction of the modified RA and traditional RA [1] was summarized in terms of “Initial burr size,” “Ablation speed,” “RA flush,” and “Burr motion” and shown in Supplementary [Supplementary-material supplementary-material-1].

### 2.3. Quantitative Coronary Angiography (QCA)

QCA was performed by standard methods and definitions using CAAS software version 5.9.1. (Pie Medical Imaging, Maastricht, the Netherlands) [16]. Angiographic success for RA + stenting was defined as (1) successful stent delivery and expansion with ≤20% in-stent residual stenosis of the target lesion [8] and (2) residual stenosis was <50% for RA + POBA and (3) <30% for RA + DCB; Meanwhile, no major adverse clinical sequelae (such as death, myocardial infarction, or emergency bypass surgery) were observed in the presence of thrombolysis with myocardial infarction flow grade 3 and no perforation [9, 17].

### 2.4. Endpoints

Early major adverse cardiac events (MACE) were the combination of all-cause death and the first occurrence of target lesion revascularization (TLR) during hospitalization and 30 days after PCI. Midterm MACE included 12-month and 24-month rates of all-cause death, cardiac death, the first occurrence of hospitalization due to heart failure, definite stent thrombosis, and TLR. TLR was defined as repeat revascularization within 5 mm proximal or distal to the target lesion or coronary artery bypass graft surgery (CABG) of the lesion in the same epicardial vessel.

### 2.5. Statistical Analysis

All analyses were performed using SPSS software version 24.0 (IBM Corp., Armonk, NY, USA). Continuous variables with abnormal distribution were presented as median (interquartile range, IQR). Categorical variables were presented as proportions (%). Clinical event rates were calculated using the Kaplan–Meier analysis, and the differences between groups were assessed with a log-rank test. Hazard ratios (HR) and 95% confidence intervals (CI) were calculated using the Cox proportional hazards model, with assumptions of proportional hazards based on the Schoenfeld residuals. Collinearity diagnosis was performed using the Spearman correlation and Belsley's criterion. For clinically similar variables, we selected the variable that we considered to be more clinically relevant. Basing on the literature [13, 18] and clinically relevance, 13 candidate variables including demographic characteristics (age), life behavior (current smoker), medical situation (hypertension, diabetes, hyperlipidemia, hemodialysis, and left ventricular ejection fraction (LVEF)), lesion characteristics (reference vessel diameter and lesion length), and treatment (PCI strategy, maximum burr/artery ratio, and minimal lumen diameter (MLD) after PCI and statin used) were screened for independent risk factors for MACE and TLR within 2 years in univariate regression. Variables with *P* < 0.10 in univariate Cox models were entered into a multivariable Cox model. All *P* values were two-sided and not adjusted for multiple testing. *P* values <0.05 were regarded as statistically significant.

## 3. Results

### 3.1. Clinical and Angiographic Baseline Characteristics

During the study period, among 1374 patients treated with RA, excluding 205 lost to follow-up, 1169 patients with 1453 lesions were included. The median age was 75 (IQR: 69–81) years, and 32.1% of patients were aged >80 years (Table 1). The prevalence of diabetes mellitus was 44.6%, old myocardial infarction 23.2%, and dialysis for end-stage renal failure 11.7%. As shown in Supplementary [Supplementary-material supplementary-material-1], there were 83.0% and 17.0% cases of de novo and in-stent restenosis (ISR) lesions, respectively. Forty (3.4% of all) cases are affected with left main trunk (LMT), 660 (56.5%) with left anterior descending artery (LAD), 189 (16.2%) with left circumflex coronary (LCX), and 298 (25.5%) with right coronary artery (RCA). In terms of the extent of disease, 64.9% of all cases had single, 25.2% double, and 9.8% triple vessel disease. Complex lesion characteristics such as bifurcation, ostial, or CTO lesions were presented in 54.7%, 42.3%, and 5.0% of patients, respectively. Of note, the American Heart Association type B2/C lesions proportion was 99.9% (16.2% and 83.7%, respectively).

### 3.2. Procedural Detailed Information

The success rate of angiography was 97.8%. QCA data were available for 1056 cases (89.7%). Pre-QCA demonstrated that the reference vessel diameter was 2.72 (2.37–3.20) mm, lesion length 30.67 (22.78–39.91) mm, and the proportion of lesions with angulation >45° (moderate/excessive) was 52.9%. The other cases were unmeasurable because of severe stenotic lesions without clear visualization of contrast filling. As shown in Supplementary [Supplementary-material supplementary-material-1], most procedures were performed via RA + DES or RA + POBA (68.3% and 26.7%). The 7-F/8F guiding catheter was used in 51.0% cases (596/1169), and femoral approach was performed in 47.0% (550/1169). The maximum burr-to-artery ratio was 0.63 (0.55–0.73), and the “step-up” technique (no. of burrs used ≥2) was used in 34.4% (402/1169) cases. The acute gain after PCI was 1.75 (1.36–2.10) mm. Two burrs were entrapped during procedures and freed by another ballooning and deep engagement of the catheter. The stent was unexpectedly underexpanded even after 1.5 mm burr rotablation in one case and was then fully expanded after bailout excimer laser coronary atherectomy with a noncompliant balloon. Most cases (91.1%, 1065/1169) were guided by imaging devices: IVUS (87.7%) and OCT (3.4%). For PCI complications, only 1.7% (20/1169) cases had coronary perforations, which were successfully sealed by POBA (7), coil (7), urgent cardiac surgery (2), or bailout metal stent (4). Transient slow flow or no reflow was observed in 0.4% (5/1169) cases.

### 3.3. In-Hospital and Midterm Clinical Outcomes

The median follow-up time was 498 days (IQR 324–727 days), and the follow-up rate was 100% and 54.8% at 12 and 24 months, respectively. Six deaths occurred during hospitalization (one died within 24 h after emergent surgery for a large coronary perforation, four died from cardiac shock, and one from pneumonia and multiple organ dysfunction) and 23 in the first 30 days (Table 2). No definite coronary acute occlusion was reported in the first month after PCI, regardless of the strategy used (RA + DES, DCB, or POBA). The cumulative incidence of MACE was 20.5% at 1-year follow-up and 26.8% at 2-year follow-up and was mainly driven by a high rate of TLR (10.3% at 1 year and 12.5% at 2 year) (Table 2). All-cause death occurred in 5.5% of patients at 1 year and 6.0% at 2 years; the majority were of cardiac origin with a mortality rate of 2.4% and 3.1%, respectively. LVEF was maintained during follow-up. Kaplan–Meier curves for MACE, all-cause death, cardiac death, and TLR are shown in Supplementary [Supplementary-material supplementary-material-1]. A total of 40.2% and 68.8% of patients underwent coronary intervention angiography and computed tomography angiogram during 24 months of follow-up, showing a relatively high late lumen loss about 1.61 mm.

Among different RA-facilitated PCI strategies, the survival curve of RA + DES started to separate at six months after PCI from that of the other two strategies. RA + DES had the lowest 2-year MACE of 14.5%, compared with the RA + DCB and RA + POBA (30.5% and 26.0%, respectively, Supplementary [Supplementary-material supplementary-material-1]).

### 3.4. Risk Factors of MACE and TLR

Univariate and multivariate analysis were used to identify the risk factors for MACE and TLR. Diabetes (adjusted hazard ratio (aHR) 1.41, 95% CI 1.08–1.85), hemodialysis (aHR 3.34, 95% CI 2.44–4.58), LVEF level (aHR 0.97, 95% CI 0.96–0.98), RA + DCB versus RA + DES (aHR 2.11, 95% CI 1.28–3.48), and RA + POBA versus RA + DES (aHR 1.58, 95% CI 1.19–2.09) were significantly related to total MACE (Table 3). Meanwhile, the aforementioned factors were also independent risk factor of total TLR (Supplementary [Supplementary-material supplementary-material-1]). Unexpectedly, maximum burr-to-artery ratio and MLD after PCI were not associated with MACE or TLR with statistical significance.

## 4. Discussion

This retrospective study presents the “real-world” utility of modified RA in the contemporary coronary angioplasty era with regular follow-up. We demonstrated that (1) the modified RA procedures were safe both in severe calcification and in ISR lesions with about 98% success rate and no definite acute occlusion occurring within the first month even with RA + POBA alone; (2) RA + DES had the best midterm outcome compared with DCB or RA + POBA; (3) the rate of MACE was mainly driven by a high but acceptable TLR rate, considering the severity of patients; and (4) diabetes, hemodialysis, low LVEF, and stent-less RA were significant predictors for MACE and TLR in patients receiving RA.

It has been >30 years since Jerome Ritchie, David Auth, and colleagues introduced RA as a technique for endovascular treatment of obstructive atherosclerotic disease [19]. Although controversies exist on the effect of RA, the process of improving the technique and strategy never stops. To our knowledge, choosing the right patient and skillful performance is vital for the success of RA. The RA procedure was performed with some modifications at our institute as follows: firstly, “Double flush” with a cocktail from guiding catheter and RA system was used since the latter flush alone may not be powerful enough to clear debris. “Double flush” increases the flushing pressure for debris passing through the coronary microvasculature, cools down the rotablator turbine, and further reduces slow/no flow occurrence, which was observed in only 0.4% (5/1169) cases in the present study, lower than previously reported [7, 18]. Secondly, choice of initial burr size was dependent on the “imaging device passage,” e.g., IVUS probe from Navifocus® WR, Intrafocus II View IT (Terumo, Japan) was <3F. If the IVUS probe could not cross the lesion, MLD was supposed to be <1.0 mm, and 1.5 mm burr was recommended. If the IVUS could cross the calcified lesion, the MLD was supposed to be >1.0 mm, and initial burr with 1.75 mm was considered to be more efficient. IVUS images could also provide details on reference vessels or target lesions for safe ablation. Since debates on adequate burr/artery ratio and the “step-up” approach are still ongoing [1, 2, 20], this concise method could be convenient and efficient. Thirdly, the strategy of “high” speed to cross, followed by “low” speed to polish, was routinely applied to warrant efficient lesion cross the lesion and sufficient plaque modification, although evidence supporting a particular speed for RA is limited. “High” ablation speed burr has more power to cross and lower deflection effect injury. However, it might increase platelet activation and thrombotic complications, which could be overcome by “double flush” with a cocktail vasodilator [21, 22]. In daily practice, the higher the speed we use, the more frequent the occurrence of severe transient bradycardia, the mechanism of which remains unclear. Intravenous atropine or mechanical maneuvers (coughing) were used to prevent the incidence and clinical consequences of transient atrioventricular blocks, and no patient needed a temporary pacemaker. After crossing, the “low” ablation speed burr polish resulted in a larger and smoother lumen with deflection motion. Crossing with a “high” speed, the risk of burr stuck and unexpected vessel injury from “low” speed would be decreased substantially. In the present study, all lesions could be successfully crossed with an acute gain of 1.75 mm, but only two burrs were entrapped (0.2%, 2/1169) and finally freed by ballooning. Fourth, the application of IVUS or OCT in the present study was mandatory (>99%). Although IVUS or OCT are frequently used to plan interventions, they are ambiguously recommended in RA procedures on severely calcified lesions [23, 24]. The current study found that these intravascular imaging devices not only provide information on the degree of calcification and vessel geometry before and after RA and stent apposition, but also enhance the safety of sufficient modification and identification of medical injury from debulking.

RA was shown to be safe for complex PCI in different trials with complication rates about 3–9% [7, 8], mainly including perforation and slow/no flow and dissection. Sakakura et al. [25] demonstrated even the lower incidence of important procedure-related complication rate as 1.3% in the J-PCI Registry, a large nationwide registration system in Japan, with each component ranging between 0.2% and 0.6%, which indicated that RA is safe and mature in skilled hands. In this study, we mainly investigated relatively high-risk subjects with old age (more than 30% patients older than 80 years) and history of previous revascularization (approximately 70%) with a large sample size. The early death rate was 2.5%, acceptable and comparable to the study reported by Okai et al. [18]. The complication rate with the modified technique was also acceptably as low as 2.2%, principally driven by coronary perforation (1.7%). The perforation rate in this study was a little higher than that of the ROTATE multicenter registry trial (1.7% vs. 1%) [7], which could be explained by the exceptionally high-risk population, older median age (75 years), more prevalence of diabetes (44.6%), hemodialysis (11.7%), and American Heart Association-type B2/C lesions (99.9%). An accurate dissection rate could not be calculated in this study, as the bailout stenting strategy for dissection could not be clearly distinguished from other stenting strategies. Nevertheless, in RA + POBA cases with a de novo lesion, the dissection rate was also low (6.8%, not shown in the Result) as previously reported [7]. The incidence of cardiac death and TLR was as low as about 2-3% and 10–12% during follow-up, respectively, relatively lower than previous data [7]. The following reasons could be attributable for the favorable results: (1) most RA procedures were performed by two experienced operators in SCVC, who could skillfully cope with the complications, consistent with the notion that the operator's technique and center experience for RA are important for patients' prognosis [6, 7]. (2) The modified RA method further reduced procedure complications and obtained sufficient ablation and better stent apposition. (3) Finally, patients were strictly followed up. These patients frequently contacted with doctors, complied with their medications and were routinely examined using a coronary computed tomography angiograph for early detection of restenosis.

The multivariable analysis revealed hemodialysis as a significant risk factor for MACE and TLR, which is consistent with previous studies [7, 26, 27]. Besides, diabetes, low LVEF, DCB, and POBA (versus DES) were also significantly associated with MACE and TLR. Biologically, chronic inflammation, oxidative stress, a bone mineral disorder from hemodialysis [28, 29], and chronic hyperglycemia from diabetes have been shown to contribute to MACE and TLR [30]. The modest association of impaired LVEF to TLR might result from the more aggressive revascularization requirement in patients with heart failure.

Among the three RA-facilitated PCI strategies, RA + DES proved to be the most effective to reduce MACE, with the metallic support of stent struts and antiproliferative drugs. Theoretically, RA and DES could act synergistically in complex lesions as RA can avert stent coating damage and DES can effectively suppress neointimal proliferation. In contrast, the ROTAXUS trial demonstrated that rotablation does not increase the efficacy of DES in calcified lesions compared with balloon dilation [8], due to the relatively low-risk patients with large reference vessel size and short period follow up [6]. In our present study, data about stenting without RA were not included, but the relatively small reference vessel size with large acute gain indicated the indispensable role of RA in a strict lesion and the favorable debulking effect of the aforementioned modified RA technique.

To our knowledge, this is the largest retrospective study analyzing the safety and efficacy of “real-world” RA in the contemporary coronary angioplasty era, especially with advanced-age patients. The follow-up rate was more than 85%, and patients were strictly evaluated (clinically and using imaging), ensuring high quality of the data. However, some limitations should be acknowledged. First, the choice of RA was based on the operators' discretion, which was more aggressive as the initial strategy than recommended. Secondly, there was no comparison between the modified RA and the standard method. Interventional coronary angiography driven by clinical symptoms would also numerically decrease MACE prevalence from the misdiagnosis of silent ischemia.

## 5. Conclusions

The present study demonstrated the modified RA technique as a safe and effective tool for high-risk patients in the contemporary PCI era, while RA + DES emerged as the most favorable interventional strategy with the lowest MACE rate. Diabetes, hemodialysis, low LVEF level, and stent-less RA strategy were independent risk factors of MACE in patients who underwent the RA procedure.

## Figures and Tables

**Table 1:** Baseline clinical characteristics of the patients (*n* = 1169).

Characteristics	No. of patients (%) or median (IQR)
Age (years)	75 (69–81)
Age >80 years	375 (32.1)
Body mass index (kg/m^2^)	23.60 (21.50–26.40)
Men	762 (65.2)
Current smoker	136 (11.6)
Diabetes mellitus	521 (44.6)
Hypertension	1018 (87.1)
Hyperlipidemia	889 (76.0)
Clinical diagnosis	
Stable angina	1132 (96.8)
Unstable angina	35 (3.0)
Non-STEMI	1 (0.1)
STEMI	1 (0.1)
Old myocardial infarction,	271 (23.2)
Previous PCI	644 (56.8)
Previous coronary artery bypass graft	139 (11.9)
Atrial fibrillation	63 (5.4)
Hemodialysis	137 (11.7)
Estimated glomerular filtration rate (ml/min·1.73 m^2^)	51 (38–62)
LVEF (%)	65 (60–69)
Medication at discharge	
*β*-blocker	250 (21.4)
Dual antiplatelet	1073 (91.8)
Statin	644 (55.1)
Eicosapentaenoic acid	98 (8.4)
Ezetimibe	43 (3.7)
Fibrates	27 (2.3)
ACEI/ARB	583 (49.9)
Calcium channel blocker	691 (59.1)
Laboratory data	
Hemoglobin (g/dL)	12.70 (11.20–13.80)
HDL-C (mg/dL)	44 (36–53)
LDL-C (mg/dL)	91 (73–113)
HbA1c (%)	6 (5.60–6.60)

ACEI, angiotensin-converting enzyme inhibitor; ARB, angiotensin II receptor blocker; HbA1c, hemoglobinA1c; HDL-C, high-density lipoprotein cholesterol; LDL-C, low-density lipoprotein cholesterol; LVEF, left ventricular ejection fraction; PCI, percutaneous coronary intervention; STEMI, ST-segment elevation myocardial infarction.

**Table 2:** Early outcome and prognosis during follow-up.

Characteristics	No. of patients (%) or median (IQR)
Early outcome (in-hospital/30 days)	
MACE	29 (2.5)
In-hospital death	6 (0.5)
30-day death	23 (2.0)
TLR	0 (0.0)
12-month follow-up	
Cumulative MACE	240 (20.5)
TLR	120 (10.3)
All-cause death	64 (5.5)
Cardiac death	28 (2.4)
Heart failure	27 (2.3)
Stent thrombosis	1 (0.1)
LVEF (%)	66 (61–69)
CAG	302 (26.0)
CTA	441 (37.9)
MLD (mm)	0.83 (0.63–1.14)
Late lumen loss (mm)	1.63 (1.08–2.19)
ISR (>50%)	131 (11.3)
24-month follow-up	
Cumulative MACE, *n* (%)	313 (26.8)
TLR	137 (12.5)
All-cause death	70 (6.0)
Cardiac death	36 (3.1)
Heart failure	69 (5.9)
Stent thrombosis	1 (0.1)
LVEF (%)	66 (61–70)
CAG	442 (40.2)
CTA	756 (68.8)
MLD (mm)	0.90 (0.75–1.35)
Late lumen loss (mm)	1.61 (1.36–2.19)
ISR (>50%)	173 (15.7)

CAG, coronary angiogram; CTA, computed tomographic angiography; ISR, in-stent restenosis; LVEF, left ventricular ejection fraction; MACE, major adverse cardiac events; MLD, minimal lumen diameter; TLR, target lesion revascularization.

**Table 3:** Predictors of major adverse cardiac events.

Variables	Univariate			Multivariable		
	cHR	95% CI	*P* value	aHR	95% CI	*P* value
Demographic characteristics						
Age (1-year increase)	0.99	0.97–1.01	0.523			
Life behavior						
Current smoker	1.02	0.70–1.49	0.923			
Medical situation						
Hypertension	1.51	0.81–2.80	0.170			
Diabetes	1.66	1.29–2.14	<0.001	1.41	1.08–1.85	0.012
Hyperlipidemia	0.819	0.62–1.09	0.165			
Hemodialysis	3.94	2.93–5.30	<0.001	3.34	2.44–4.58	<0.001
LVEF	0.97	0.96–0.98	<0.001	0.97	0.96–0.98	<0.001
Lesion characteristics						
Reference vessel diameter	1.16	0.95–1.41	0.138			
Lesion length	0.99	0.99–1.01	0.528			
Treatment						
PCI strategy						
RA + DES	Ref			Ref		
RA + DCB	2.38	1.47–3.86	<0.001	2.11	1.28–3.48	0.004
RA + POBA	1.84	1.42–2.40	<0.001	1.58	1.19–2.09	0.002
Max burr/artery ratio	1.78	0.82–3.86	0.144			
MLD after PCI	0.92	0.74–1.15	0.461			
Statin used	0.81	0.63–1.04	0.091			

aHR, adjusted hazard ratios; CI, confidence interval; cHR, crude hazard ratios; DCB, drug-coated balloon; DES, drug-eluting stent; LVEF, left ventricular ejection fraction; MLD, minimal lumen diameter; POBA, plain old balloon angioplasty; RA, rotational atherectomy.

## Data Availability

The data used to support the findings of this study are available from the corresponding author upon request.

## Supplementary Materials

Supplementary Materials
[Supplementary-material supplementary-material-1] include the comparison of traditional and modified rotational atherectomy (RA) technique, baseline lesion characteristics, PCI procedure and quantitative coronary angiography (QCA) data, and predictors of target lesion revascularization. Besides, the cumulative incidence curves for adverse events and the comparison of major adverse cardiac events among different PCI strategies are shown in the supplementary figures.
