# Supplementary material for: Reappraisal Value of a Modified Rotational Atherectomy Technique in Contemporary Coronary Angioplasty Era
Source: J Interv Cardiol. 2020 Jan 23;2020:9190702. doi: 10.1155/2020/9190702 (PMC7007738; doi:10.1155/2020/9190702)
Supplement: Supplementary file 1 [file 9190702.f1.odt]

Supplementary Table 1. Comparison of traditional and modified RA technique

|  | Traditional | Modified |
| --- | --- | --- |
| Initial burr size | Plaque modification with small burrs (1.25 mm to 1.5 mm) as initial strategy is default position. | 1)≧1.75 mm if IVUS/OCT can cross the lesion； 2)≦1.5 mm if IVUS/OCT can not cross the lesion. |
| Ablation speed | Plaque modification usually achieved at low speeds (135,000 to 180,000 rpm) to reduce risk of complications. | Pass at "high" speed (160,000–220,000 rpm); then polish at “low” speed (140,000–160,000 rpm). |
| RA flush | Rotablation cocktail with verapamil, nitrates and heparin in saline recommended | Double flush: RA-cocktail shower + intracoronary flush-cocktail from guiding catheter |
| Burr motion | Pecking motion | Cruising motion |

Supplementary Table 2. Baseline lesion characteristics of the patients (n=1169)

| Characteristics | No. patients (%) or median (IQR) |
| --- | --- |
| De novo lesion | 970 (83.0) |
| ISR lesion | 199 (17.0) |
| Target vessel |  |
| LMT | 40 (3.4) |
| LAD | 660 (56.5) |
| LCX | 189 (16.2) |
| RCA | 298 (25.5) |
| Extent of the disease |  |
| Single vessel disease | 759 (64.9) |
| Double vessel disease | 295 (25.2) |
| Triple vessel disease | 115 (9.8) |
| Bifurcation lesion | 640 (54.7) |
| Ostial lesion | 494 (42.3) |
| CTO | 59 (5.0) |
| Lesion type (AHA) |  |
| A | 0 (0) |
| B1 | 2 (0.2) |
| B2 | 189(16.2) |
| C | 978 (83.7) |
| Pre-QCA |  |
| Reference vessel diameter (mm) | 2.72 (2.37–3.20) |
| Lesion length (mm) | 30.67 (22.78–39.91) |
| Lesion angulation >45° | 618 (52.9) |

AHA, American Heart Association; CTO, chronic total occlusion; LAD, left anterior descending artery; LCX, left circumflex coronary; LMT, left main trunk; QCA, quantitative coronary angiography; RCA, right coronary artery.   

Supplementary Table 3. PCI procedure and QCA data (n=1169) 

| Characteristics | No. Patients (%) or median (IQR) |
| --- | --- |
| PCI strategy |  |
| RA+DES | 798 (68.3) |
| RA+DCB | 59 (5.0) |
| RA+POBA | 312 (26.7) |
| Guiding catheter ≥7 (F) | 596 (51.0) |
| Femoral approach | 550 (47.0) |
| Fluoroscopy time (min) | 25.15(18.73–34.08) |
| Contrast volume (ml) | 188(157–227) |
| Maximum stent diameter (mm) | 3 (2.50–3.50) |
| Maximum burr size (mm) | 1.75 (1.50–1.75) |
| Maximum balloon diameter (mm) | 3 (2.50–3.50) |
| Maximum balloon pressure (atm) | 20 (14–24) |
| Maximum burr-to-artery ratio | 0.63 (0.55–0.73) |
| Maximum balloon-to-artery ratio | 1.11 (1–1.28) |
| Maximum stent-to-artery ratio | 0.97 (0–1.10) |
| Minimum rotational speed | 140000 (120000–160000) |
| Total stents length (mm) | 38.00 (24 –38) |
| No. of burrs used (≥2) | 402 (34.4) |
| No. of stents used | 1 (0–1) |
| Immediate QCA |  |
| MLD before PCI (mm) | 0.95 (0.70–1.21) |
| % DS before PCI | 63 (56–72) |
| MLD after PCI (mm) | 2.73 (2.31–3.19) |
| % DS after PCI | 2.00 (-7.00–13.00) |
| Acute gain (mm) | 1.75 (1.36–2.10) |
| Image device | 1065 (91.1) |
| PCI complications | 26 (2.2) |
| Perforation | 20 (1.7) |
| Slow/no flow | 5 (0.4) |
| Burr trap | 1 (0.1) |

BMS, bare metal stent; DCB, drug-coated balloon; DES, drug-eluting stent; MLD, minimal lumen diameter; POBA, plain old balloon angioplasty; QCA, quantitative coronary angiography; RA, rota­tional atherectomy; %DS, percent diameter ste­nosis.

Supplementary Table 4. Predictors of target lesion revascularization

| Variables | Univariate |  | Multivariable |
| --- | --- | --- | --- |
| cHR | 95% CI | P-value |  | aHR | 95% CI | P-value |
| Demographic characteristics |
| Age(1-year increase) | 0.98 | 0.96-1.00 | 0.056 |  |  |  |  |
| Life behavior |
| Current smoker | 1.33 | 0.83–2.11 | 0.237 |  |  |  |  |
| Medical situation |  |  |  |  |  |  |  |
| Hypertension | 1.60 | 0.81-3.16 | 0.181 |  |  |  |  |
| Diabetes | 1.98 | 1.40–2.81 | <0.001 |  | 1.70 | 1.18–2.47 | 0.005 |
| Hyperlipidemia | 1.11 | 0.73–1.67 | 0.627 |  |  |  |  |
| Hemodialysis | 3.68 | 2.45–5.52 | <0.001 |  | 3.03 | 1.96–4.68 | <0.001 |
| LVEF | 0.97 | 0.96–0.99 | 0.001 |  | 0.99 | 0.97–1.01 | 0.066 |
| Lesion characteristics |  |  |  |  |  |  |  |
| Reference vessel diameter | 1.36 | 1.05-1.76 | 0.021 |  |  |  |  |
| Lesion length | 0.99 | 0.99–1.01 | 0.907 |  |  |  |  |
| Treatment |  |  |  |  |  |  |  |
| PCI strategy |  |  |  |  |  |  |  |
| RA+DES (Ref) | 1.00 |  |  |  | 1.00 |  |  |
| RA+DCB | 4.81 | 2.74–8.45 | <0.001 |  | 4.53 | 2.54–8.06 | <0.001 |
| RA+POBA | 3.17 | 2.20–4.58 | <0.001 |  | 2.76 | 1.87–4.08 | <0.001 |
| Maximum burr/artery ratio | 2.57 | 0.95–6.99 | 0. 064 |  |  |  |  |
| MLD after PCI | 0.84 | 0.62–1.12 | 0. 237 |  |  |  |  |
| Statin used | 1.33 | 0.94–1.90 | 0.112 |  |  |  |  |

aHR, adjusted hazard ratios; CI, confidence interval; cHR, crude hazard ratios; DCB, drug-coated balloon; DES, drug-eluting stent; LVEF, left ventricular ejection fraction; MLD, minimal lumen diameter; POBA, plain old balloon angioplasty; RA, rota­tional atherectomy.

 

Supplementary Figure 1. Cumulative incidence curves for (A) major adverse cardiac events (MACE), (B) all-cause death, (C) target lesion revascularization (TLR) and (D) cardiac death.

Supplementary Figure 2. Comparison of MACE among different PCI strategies. DCB, drug-coated balloon; DES, drug-eluting stent; MACE, major adverse cardiac events; POBA, plain old balloon angioplasty; RA, rota­tional atherectomy.
